# Mass cytometry reveals immune atlas of urothelial carcinoma

**DOI:** 10.1186/s12885-022-09788-7

**Published:** 2022-06-20

**Authors:** Qing Zhang, Wenlong Zhang, Tingsheng Lin, Wenfeng Lu, Xin He, Yuanzhen Ding, Wei Chen, Wenli Diao, Meng Ding, Pingping Shen, Hongqian Guo

**Affiliations:** 1 Department of Urology, Affiliated Drum Tower Hospital, Medical School of Nanjing University, Institute of Urology, Nanjing University, 321 Zhongshan Road, Nanjing, 210008 Jiangsu China; 2 Department of Urology, Drum Tower Hospital Clinical College of Nanjing Medical University, 321 Zhongshan Road, Nanjing, 210008 Jiangsu China; 3 State Key Laboratory of Pharmaceutical Biotechnology, Department of Urology, School of Life Science, Nanjing Drum Tower Hospital, The Affiliated Hospital of Nanjing University Medical School, Nanjing University, Nanjing, 210023 Jiangsu China

**Keywords:** Urothelial carcinoma, Immunotherapy, Immune profiling, Mass cytometry, Microenvironment

## Abstract

Immunotherapy has emerged as a robust clinical strategy for cancer treatment. PD1/PD-L1 inhibitors have been used as second-line therapy for urothelial carcinoma due to the high tumor mutational burden. Despite the efficacy of the treatment is significant, the response rate is still poor. The tumor immune microenvironment plays a key role in the regulation of immunotherapeutic efficacy. However, a comprehensive understanding of the intricate microenvironment in clinical samples remains unclear. To obtain detailed systematic tumor immune profile, we performed an in-depth immunoassay on 12 human urothelial carcinoma tissue samples and 14 paratumor tissue samples using mass cytometry. Among the large number of cells assayed, we identified 71 T-cell phenotypes, 30 tumor-associated macrophage phenotypes. T cell marker expression profiles showed that almost all T cells in the tumor tissue were in a state of exhaustion. CD38 expression on tumor-associated macrophages (TAMs) was significantly higher than PDL1, and CD38^+^ TAMs were closely associated with immunosuppression. CD38 may be a more suitable target for immunotherapy in urothelial carcinoma compared to PD1/PDL1. This single-cell analysis of clinical samples expands our insights into the immune microenvironment of urothelial carcinoma and reveals potential biomarkers and targets for immunotherapy development.

**Supplementary Information:**

The online version contains supplementary material available at 10.1186/s12885-022-09788-7.

## Introduction

Urothelial carcinoma (UC) as a common genitourinary cancer, accounting for 4.5% of all new cancer cases and 3% of cancer-related deaths in 2020, is the sixth most common type of cancer [1]. Urothelial carcinoma develops primarily in the bladder, but may also occur in other parts of the urethra, such as the kidneys, ureters, and urethra. Based on the depth of bladder wall infiltration, bladder cancer is clinically classified into muscle-invasive (MI-BC) and non-muscle-invasive (NMI-BC). Of these, NMI-BC is usually multifocal, accounting for about 80%, and is treated with the intent of cure, while MI-BC accounts for about 20% and is characterized by a high incidence of distant metastases even after total cystectomy and systemic chemotherapy, with a five-year survival rate of less than 5% [2]. Currently, standard radical surgical treatment supplemented with intravenous chemotherapy, such as gemcitabine & cisplatin (GC) regimen, is commonly used in clinical practice, but the prognosis of patients is unsatisfactory and shows a tendency to metastatic progression [3, 4].

Immunotherapy, in particular immune checkpoint inhibitors (ICI), has emerged as a therapeutic breakthrough and has produced significant clinical responses in patients with several solid tumors [5]. Because of the high mutational burden, bladder cancer ranks as the 3rd most common type of malignancy and is considered suitable for ICI treatment [6]. The use of checkpoint inhibitors has shown an exciting response to metastatic UC that has failed with conventional platinum-based chemotherapy. Atezolizumab [7], an inhibitor of PD-L1, is the first checkpoint inhibitor to be approved for bladder cancer. In a phase I trial of 68 previously treated patients with advanced bladder cancer, the objective remission rate (ORR) for atezolizumab ranged from 11 to 43% [8]. Although ICI has achieved some impressive efficacy in the treatment of bladder cancer, overall only 20–25% of treated patients with advanced disease have responded [9]. Higher PD-L1 expression tends to promote response to ICI therapy, but patients with low PD-L1 expression may still respond [10]. Therefore, it is necessary to further elucidate the immune regulatory mechanism of urothelial carcinoma and provide new ideas for immunotherapy.

The tumor is a complicated tissue, and different types of non-tumor cells surrounding tumor cells together constitute the tumor microenvironment, such as fibroblasts, immune cells, endothelial cells, macrophages [11]. These non-tumor cells are essential for tumor progression. The imbalance of immune homeostasis in the tumor microenvironment (TME) is an essential feature of tumors, which leads to suppression of the infiltration, survival, and activation of effector cells with immune recognition and tumor-killing functions. Under the influence of continuous stimulation of tumor antigens and various factors in TME (such as the immunosuppressive cytokines, immunosuppressive cells, hypoxia, lack of nutrition, acidic physical and chemical environment), the function of CD8^+^ T cell gradually decreases, manifested by reduced proliferation ability, susceptibility to apoptosis, and decreased levels of secreted effector cytokines (such as IL-2, IFN-γ, TNF-α), which is called “T cell exhaustion” [12]. As the central regulator of tumor immunity, tumor -associated macrophages (TAMs) account for the highest proportion of all immune cells in the tumor microenvironment. The phenotypic and functional status of TAMs is tightly correlated with the status of the tumor immune microenvironment, thus targeting TAMs is a prospective therapeutic strategy [13]. However, the heterogeneity of TAMs has been one of the greatest challenges of this therapeutic strategy [14].

The phenotypes of T cells and TAMs have been recognized as important biomarkers in immunotherapy [15]. Immune checkpoint blocking therapy can effectively overcome T cell exhaustion in various cancer types, while some studies have shown that TAMs play an important role in ICI therapy resistance. The lack of definition of TAM and T cell phenotypes in human tumor tissue and their relationship in TME has greatly limited the widespread use of TAM and T cells as biomarkers and drug targets [16]. The application of technology at the single-cell level provides researchers with a new perspective on the complexity of TME. Single-cell mass spectrometry flow cytometry (CyTOF) uses the principle of mass spectrometry to detect over 50 indicators simultaneously at the single-cell level [17]. CyTOF allows high-throughput multiparametric detection of large numbers of cells, which in turn enables precise immunophenotyping of cell populations [18], and therefore can be the preferential method for analyzing the phenotypic diversity of cells in TME [19, 20].

In this study, we present an immune profile of urothelial carcinoma using CyTOF from tumor samples of 14 patients with urothelial carcinoma. In contrast to the achievements of immunotherapy in tumors such as melanoma and lung cancer, successful immunotherapeutic approaches for bladder cancer remain inadequate and the efficacy of treatments is highly variable between patients. Exploring new tumor immunotherapies and finding new targets and effective biomarkers through a deep understanding of the tumor immune microenvironment in bladder cancer is of great importance for the diagnosis and treatment of bladder cancer. We utilized unsupervised computational approaches to uncover the unsurpassed phenotypic complexity of TMEs in samples from patients with urothelial carcinoma. Our data exhibited a diversity of T cell immunosuppressive phenotypes and further suggested connections between TAM phenotypes and CD8^+^ immunosuppressive T cell populations.

## Methods

### Preparation of tumor tissue single-cell suspensions

Tumor specimens were obtained by surgical resection of patients diagnosed with urothelial carcinoma. Combine the diagnostic imaging features to determine the location of the tumor site. Select intact, erythematous tumors for tumor samples. Take the tissue at least 2 cm away from the tumor site as the paratumor samples, and the pathologist confirmed that the sample was cancer-free by fast freezing pathology. Tumor samples and para-tumor samples were paired. Supplementary Table 1 summarizes the characteristics of the patients. Tumor specimens were first mechanically cut into small pieces and then next enzymatically digested (RPMI 1640 containing 2 mg/mL collagenase, 250 µg/mL hyaluronidase, 20 µg/mL deoxyribonuclease I) to generate single-cell suspensions. An average of 1–3 × 10^6^ cells were prepared and stained separately for each sample.

### Cell staining for mass cytometry

Cells were suspended in flow cytometry buffer (1xPBS + 0.5%BSA, from Sigma-Aldrich) and stained for viability using 250 nM 194Pt cisplatin (Fluidigm, San Francisco, CA). Cells were blocked by Fc receptor blocking solution (BioLegend, San Diego, CA). Antibody cocktail is incubated with cells for 30 min on the ice to stain the markers on the cell membrane. Cells were washed twice with flow cytometry buffer and use 200 μL insertion solution (Maxpar Fix and Perm Buffer containing 250 nM 191/193Ir, Fluidigm) to fix the cells overnight. After fixation, cells were washed once with flow cytometry buffer and then use perm buffer (eBioscience) to make them permeable and stain on ice for 30 min to obtain intracellular markers. To provide for reduced inter-sample variability in antibody staining, as well as to minimize the effect of variations in instrument sensitivity, we used mass labeled cell barcodes (MCB) for each sample. Resuspend the cells in deionized water containing 20% (V/V) EQ beads (Fluidigm), then filter the cells through sterile 40-mm cell strainers.

### Mass cytometry (CyTOF)

A summary of the 42 mass-conjugated antibodies used in the study to identify immune cells is shown in Supplementary Table 2. For mass cytometry acquisition, cells were resuspended in ddH_2_0 containing EQ beads (Fluidigm). All samples were assayed on a Helio3 CyTOF mass flow cytometer (Fluidigm), and the data obtained were normalized using the bead-based normalizer [21]. CyTOF analysis was conducted by PLTTech Inc. (Hangzhou, China) following the previously stated protocol [22]. All 42 immune cell markers were used to cluster and visualize. Cells were clustered based on the X-shift algorithm [23]. 100,000 cells per sample were randomly selected for clustering, and the cells with less than 100,000 were all input. All marker expressions (raw data) were arcsinH-transformed with a cofactor of 5 (counts_transf = asinh(x/5)). For visualization of high-dimensional data on two dimensions, we analyzed 10,000 randomly selected cells from each sample using the R package cytofkit by the t-SNE algorithm [24]. Immune cell subpopulations were identified based on the median value of specific markers expression in the hierarchical clusters. For marker expression level visualization on t-SNE plots, the expression was normalized.

### Immunohistochemistry

We selected formalin-fixed paraffin-embedded (FFPE) sections of bladder cancer tissue. Sections were dewaxed and hydrated and autoclaved for 10 min using 1 X citrate buffer for antigen retrieval. Slides were treated with 3% H_2_O_2_ for 15 min to inactivate endogenous peroxidase and blocked using 5% BSA/PBS/0.1% Triton X-100 for 1 h. Primary antibodies and HRP-conjugated secondary antibodies were diluted in 2% BSA/PBS, for which primary antibodies were incubated overnight at 4℃, and secondary antibodies were incubated for 1 h at room temperature. Bound antibodies are visualized with diaminobenzidine (DAB).

### Differential analysis of single-cell sequencing data

The single-cell RNA sequencing data of bladder cancer were downloaded from the SRA database (BioProject PRJNA662018). CD38^+^CD163^+^C1QA^+^cells defined as CD38^+^ TAMs, CD38^−^CD163^+^C1QA^+^ cells defined as CD38^−^ TAMs. The differentially expressed genes (DEGs) between CD38^+^ TAMs and CD38^−^ TAMs for further GO enrichment analysis and Pathway analysis.

### Tumor model and injection of anti-CD38 antibody

The establishment of orthotopic bladder cancer model: Female C57BL/6 mice (8 weeks old, weight 18–20 g) were purchased from the Model Animals Research Center, Nanjing University. All animal experiments were approved by the Institutional Animal Care Committee of Jiangsu Province and the Ethics Committee of Nanjing Drum Tower Hospital, Medical school of Nanjing University. An orthotopic bladder cancer model was established in C57BL/6 mice with a minimally invasive method based on our previously published article [25]. Briefly, we made a small incision in the skin of the lower abdomen. The bladder was found and MB-49 cells (2 × 10^5^) were injected into the bladder wall using a 1 ml syringe. For antibody treatment experiments, anti-CD38 monoclonal antibody or IgG2a isotype control antibody were administered intraperitoneally every 48 h starting on day 7 after injection. The mice were humanely euthanized on day 22, and tumor tissues were harvested and weighed.

### Statistics

Comparisons between the two groups were assessed using paired Student’s t-tests. The rationale for using these tests was based on an assessment of the normality and variance of the data distribution. Data were analyzed using Graphpad Prism (v7). Correlation analyses between cluster percentages were performed by computing Spearman’s rank-based correlation coefficient in GraphPad Prism. Differences were considered significant when the p-value was < 0.05.

## Results

### Single-cell atlas of the urothelial carcinoma immune microenvironment

**Fig. 1:**
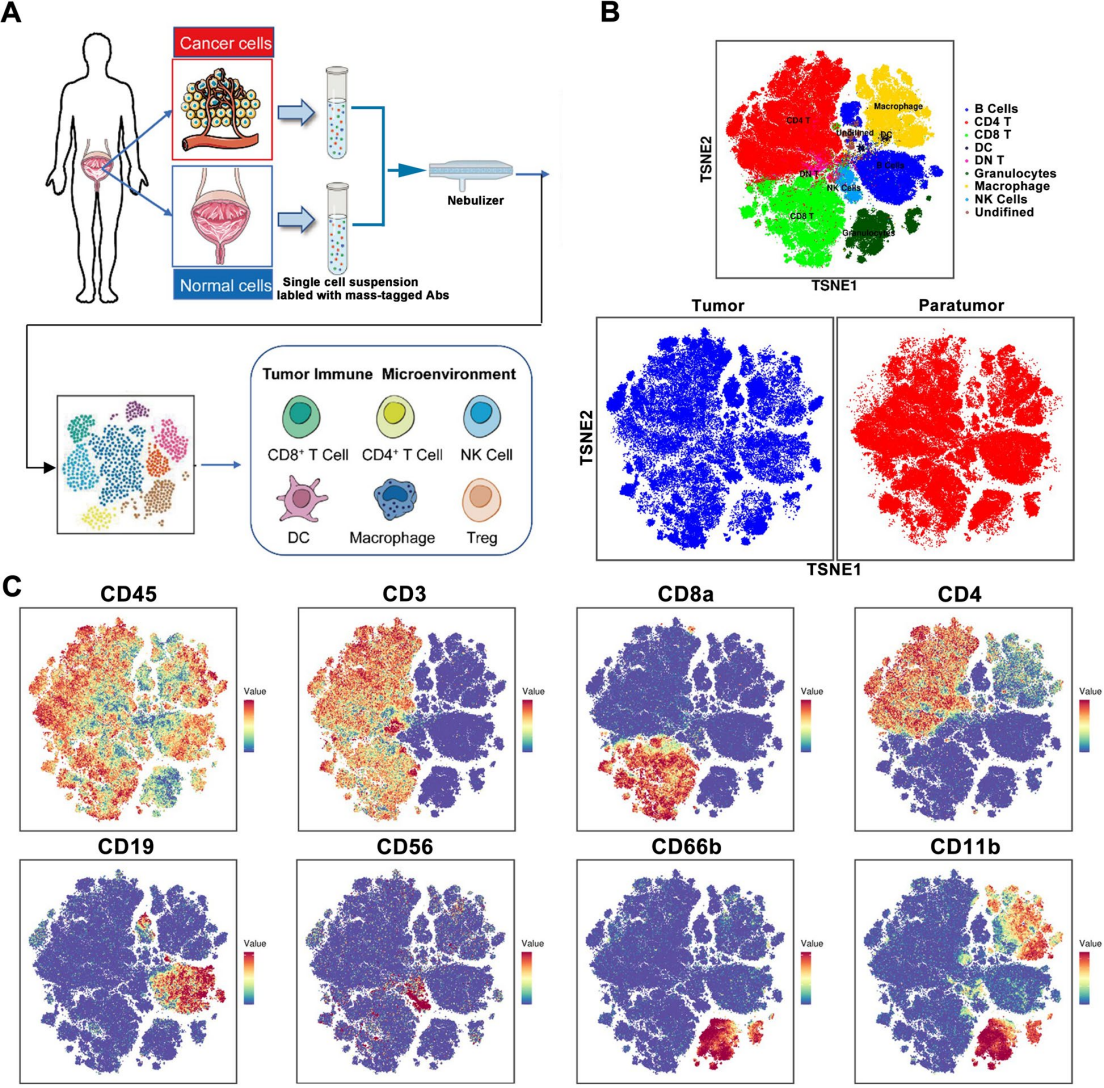
Single-cell atlas of the urothelial carcinoma immune microenvironment. **A** Workflow for immunophenotyping of urothelial carcinoma using mass Cytometry. **B** TSNE visualization shows the immune profile of tumor tissues and paratumor tissues, with the distribution of immune cell subpopulations in different colors. **C** Normalized expression of markers with coloring indicated on the t-SNE map

We performed mass cytometry analysis for 12 tumor specimens from patients with a different subtype of urothelial carcinoma (including 4 ureteral cancer, 3 renal pelvic cancer, and 5 bladder cancer) and 14 paired para-cancer tissue (Fig. 1A, Supplementary Table 1). All tissue samples are generated into single-cell suspensions by an automated system, and the cells are subsequently stained by a mixture of 42 metal-conjugated antibodies and analyzed by mass cytometry (Supplementary Table 2). Gating strategies were shown in Supplementary Figure S1. These antibodies for the identification of tumor-associated macrophages (TAMs), T cells, B cells, natural killer cells, dendritic cells, plasma cells, and granulocytes (Fig. 1B and C). To make sure of high-quality data, we confirmed the similarity of marker expression between replicate samples as well as the percentage of each immune cell subpopulation. Correction for minimal spillover between detection channels was performed by a bead-based compensation workflow.

### Immune landscape in urothelial carcinoma

By generating a two-dimensional map of the data using the dimensionality reduction algorithm t-SNE for each tumor sample, we obtained a comprehensive view of the immune profiling. To classify cells by different phenotypes, we used a digital image clustering algorithm to cluster and analyze single-cell data by computing the adjacency of cell phenotypes in a high-dimensional space. This analysis defined the major immune cell types that were involved in the T-cell and TAM data.

**Fig. 2:**
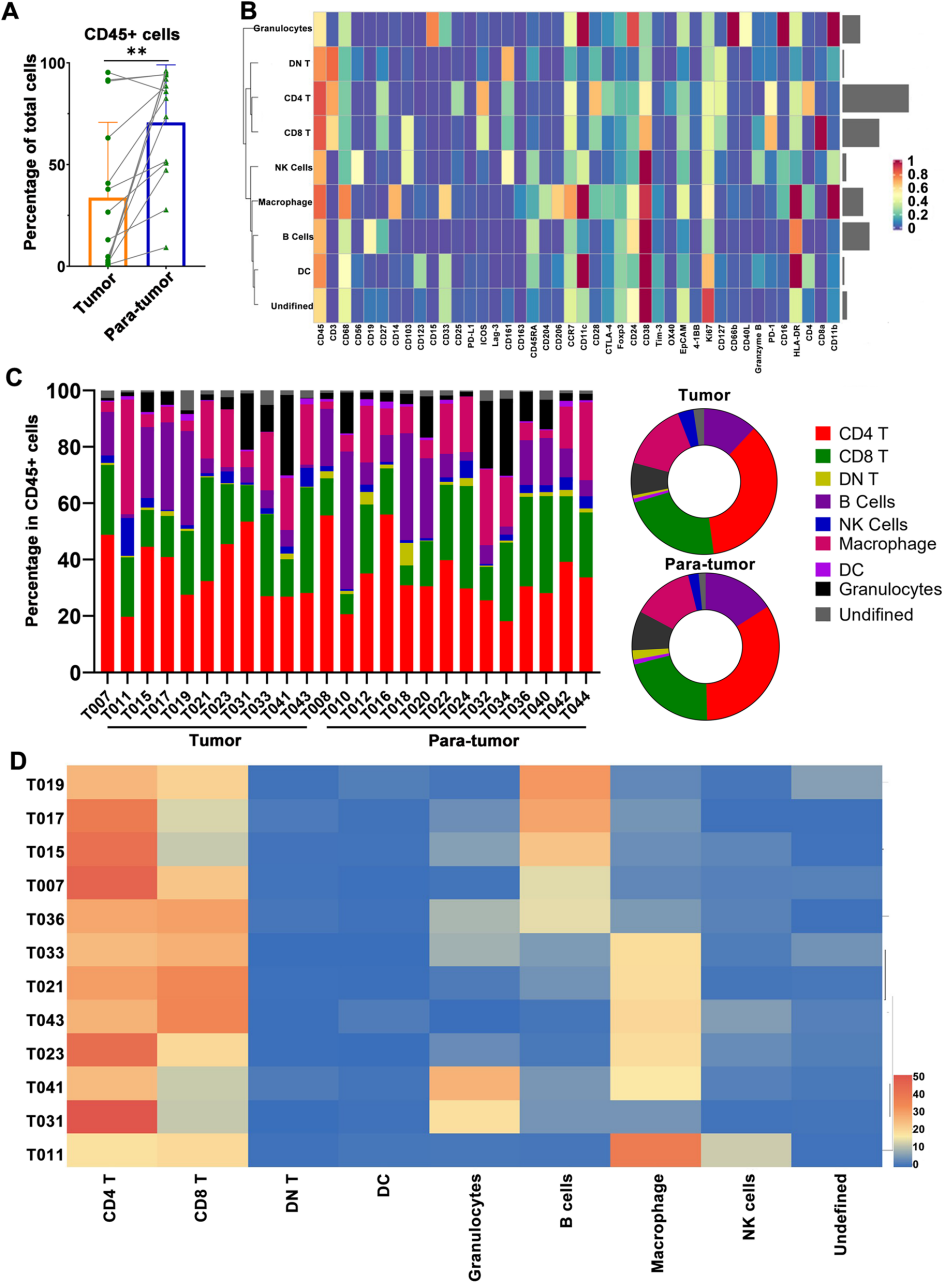
Immune landscape in urothelial carcinoma. **A** Percentage of CD45^+^ cells in tumor samples and paratumor samples. **B** The normalized expression of 42 markers is shown in Heatmap. **C** The population percentage of all immune cell types in tumor tissues and paratumor tissues. **D** Heatmap showing the population percentage of all immune cell types in the tumor tissue of each patient

Compared to the adjacent tissues, the infiltration of CD45^+^ leukocytes in tumor tissues was significantly reduced (Fig. 2A), indicating that bladder cancer is a “cold” tumor with a weak immune response. As the major immune cell population, T cells account for an average of 50% of the immune microenvironment in urothelial carcinoma (Fig. 2B and C). The next most frequent cell populations are myeloid cells and B cells. We further cluster the patients based on immunophenotyping, and the results show that tumor samples were divided into two groups: one is macrophage-enriched, the other is B-cell-enriched (Fig. 2D).

### T cell characteristics of urothelial carcinoma

**Fig. 3:**
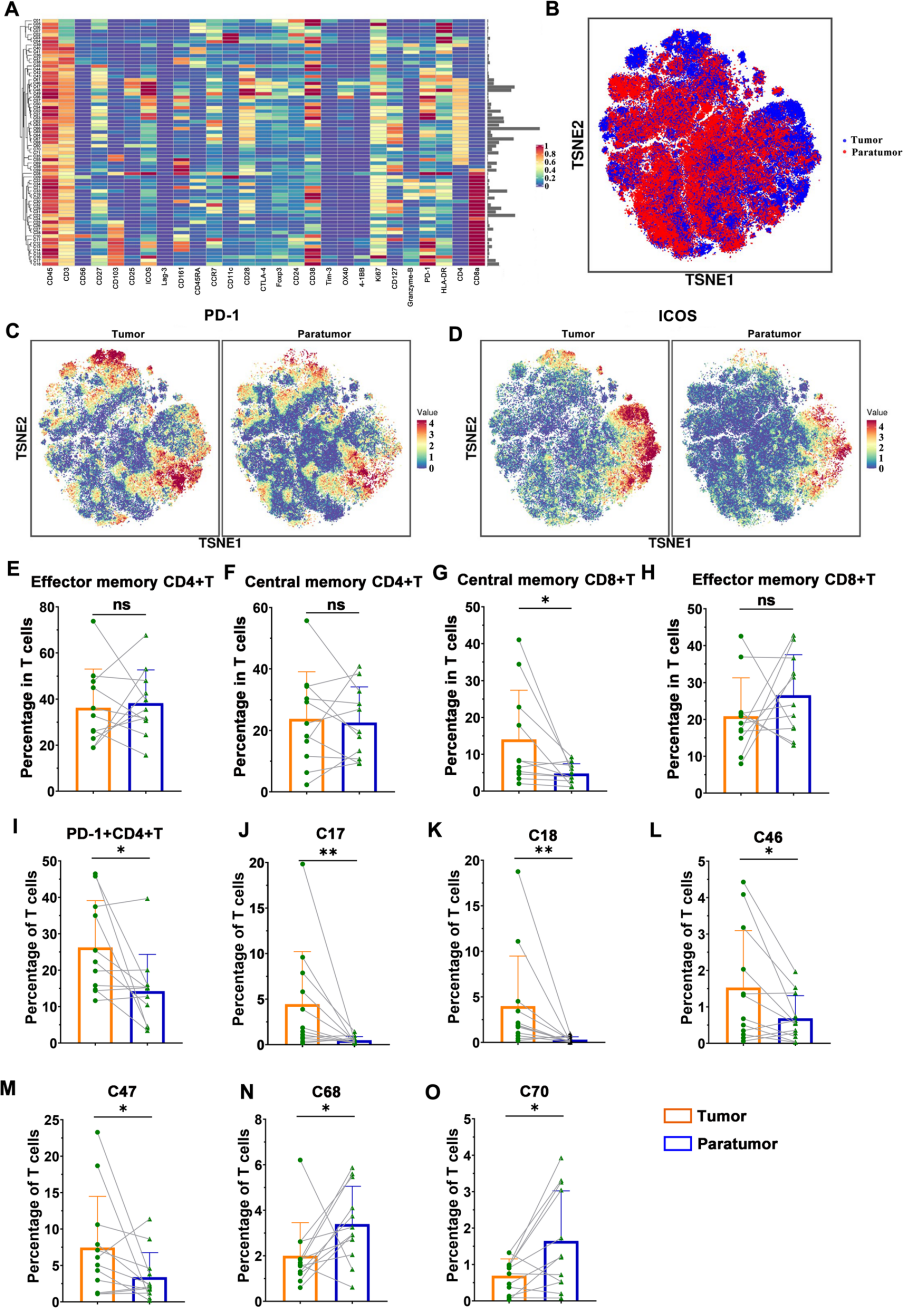
T cell characteristics of urothelial carcinoma. **A** Heatmap showing normalized expression profile of the 71 T cell clusters. **B** TSNE visualization showing the distribution of T cell clusters in tumor tissue and paratumor tissue. **C**-**D** TSNE visualization showing the normalized expression of PD-1 and ICOS. **E**-**I** Percentage of effector memory CD4^+^ T cell, central memory CD4^+^ T cell, central memory CD8^+^ T cell, effector memory CD8^+^ T cell, and PD-1^+^CD4^+^ T cell in tumor samples and paratumor samples. **J**-**O** Percentage of indicated T cell clusters in tumor tissues and paratumor tissues

T-SNE and X-shift identified dominant phenotypes when the data had a plentiful population structure. A separate analysis of these dominant phenotypes revealed a finer granularity of structure. For detailed mapping of cell phenotypes, additional image analysis was performed on each subset of T cells and TAMs which were defined by the pre-analysis. In a heat map, the expression profile of T cell clusters is shown (Fig. 3A and B). Heterogeneity of marker level was evaluated by t-SNE at a single-cell level (merge diagram). This method identified 71 T-cell phenotypes, including 28 CD4^+^T cell phenotypes, 28 CD8^+^T cell phenotypes, and 15 double-negative T cell phenotypes. The expression of PD-1, ICOS, and CCR7 on T cells in tumor tissues was significantly higher than that of T cells in paratumor tissues (Fig. 3C, D and Supplementary Fig. 2A-C). Most T cell clusters have memory phenotype (CD45RA^−^, including effector memory phenotype and central memory phenotype). There is no significant difference in the average percentage of CD4^+^ memory T cells in the T cell compartment between adjacent cancer and cancer tissues (Fig. 3E and F). The proportion of CD8^+^ central memory T cells (CD45RA^−^CCR7^+^CD8^+^) was increased in tumor tissues, while CD8^+^ effector memory T cells (CD45RA^−^CCR7^−^ CD8^+^) were unchanged (Fig. 3G and H). In cancer tissues, the CD4^+^T cell cluster (C66) had the highest percentage, reaching 8.74% (Supplementary Fig. 2D).

The coexpression of activation molecules (HLA-DR, CD38) and co-inhibitory molecules (PD-1, CTLA-4) represents the T cell exhaustion, the results showed that almost all clusters had exhaustion phenotype in the immune microenvironment of urothelial carcinoma tissues. In addition, CD38 was observed to be more widely expressed on T cells than on PD-1. Studies have reported that the ratio of PD-1^+^CD38^+^T cells is related to poor prognosis, and CD38-mediated immunosuppression is one of the mechanisms for tumor cells to escape PD-1/PD-L1 block [26]. Our data showed that T cells expressing PD-1 in urothelial tumor specimens have an unexpected phenotypic diversity. Compared with the adjacent tissues, the percentage of PD-1^+^CD4^+^T cells in cancer tissues was significantly increased (Fig. 3I). Among the 71 T cell clusters, only the percentage of C17, C18, C46, C47, C68, and C70 are significantly different in the adjacent tissues from cancer tissues, among which CD4^+^ T cell clusters (C46, C47) and CD8^+^ T cell clusters (C17, C18) were enriched in cancer tissues, and CD4^+^ T cell clusters (C68, C70) were reduced in cancer tissues (Fig. 3J-O). C68 cluster was a CD4^+^ central memory T cell, which was characterized by CD27^−^CD28^+^ICOS^−^CD127^+^HLA-DR^−^Ki67^low^, while the C70 cluster was a CD4^+^ effector memory T cell, which was characterized by CD27^−^CD28^low^ICOS^−^CD127^+^HLA-DR^−^Ki67^low^. The proportion of these two clusters in the inactive state was relatively low in the tumor, probably due to the fact that activation causes them to lose some of their functions and unable to exert their effector capacity. C17 cluster and C18 cluster were CD8^+^ effector memory T cells, which were characterized by CD103^+^ICOS^+^PD-1^+^CD38^+^CD27^+^CD161^−^HLA-DR^low^. The phenotype indicates that the two clusters are exhausted tissue-resident T cells. There is a strong correlation between the C17 cluster and the C18 cluster (Supplementary Fig. 2E). The C46 and C47 subsets belong to regulatory T cells (Treg), which were identified according to the expression of CD4, FOXP3, CD25, and CTLA-4. Compared with adjacent tissues, Treg was enriched in cancer tissues (Supplementary Fig. 2F), which indicated that the immunosuppressive microenvironment of cancer tissues. C46 cluster was a typical Treg cell population expressing a large number of active state markers and inhibitory markers.

### TAM characteristics of urothelial carcinoma

**Fig. 4:**
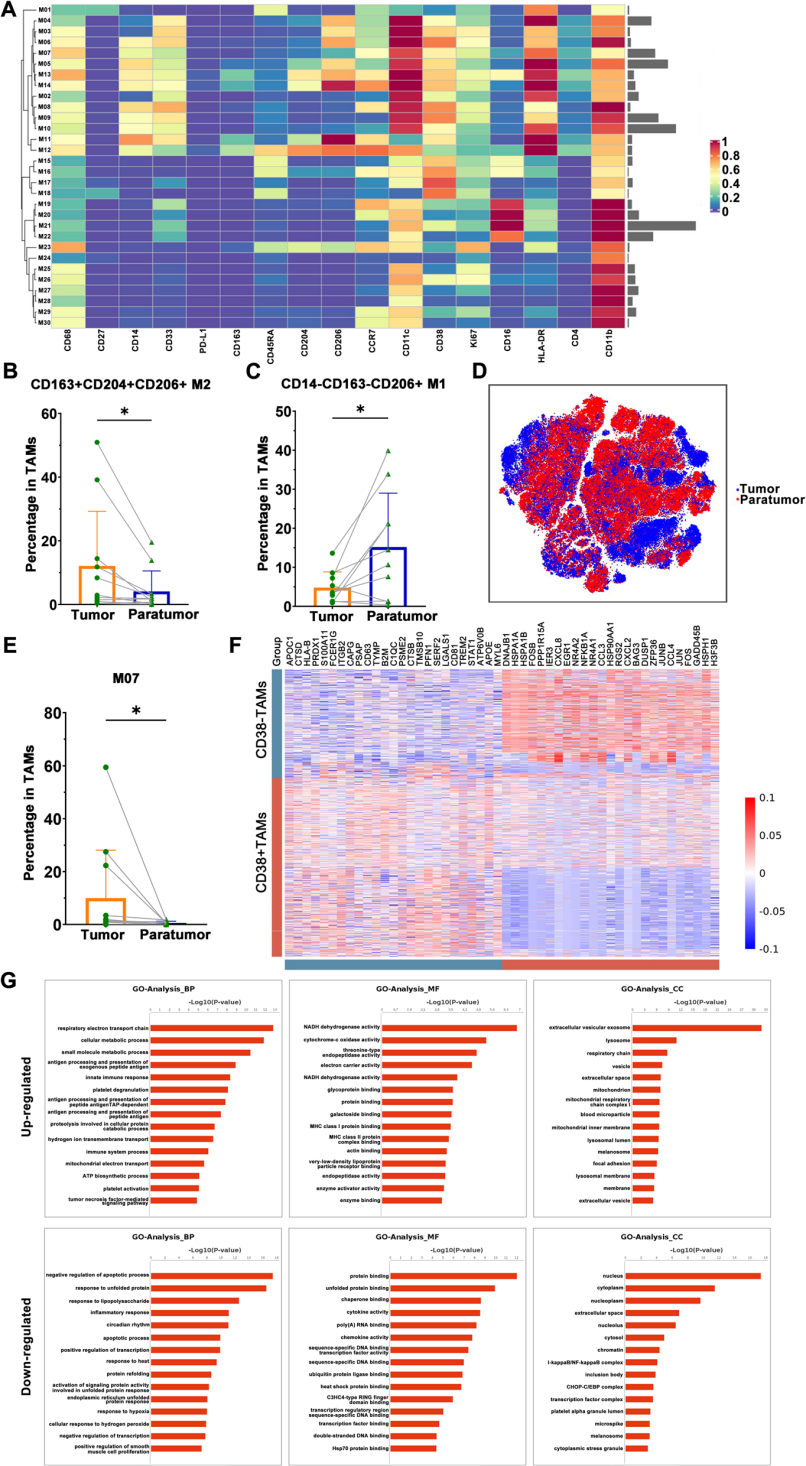
TAM characteristics of urothelial carcinoma. **A** Heatmap showing normalized expression profile of the 30 TAM clusters. **B**-**C** Percentage of CD163^+^CD204^+^CD206^+^ cells and CD14^−^CD163^−^CD206^+^ cells in tumor tissues and paratumor tissues. **D** TSNE visualization showing the distribution of TAM clusters in tumor tissue and paratumor tissue. **E** Percentage of M07 TAM clusters in tumor tissues and paratumor tissues. **F** Heatmap showing differentially expressed genes (DEGs) of CD38^+^ TAMs和CD38^−^ TAMs in single-cell sequencing data published by *Zhaohui Chen*(BioProject PRJNA662018 in SRA datasets). **G** GO enrichment analysis on the upregulated and downregulated DEGs of CD38^+^ TAMs and CD38^−^ TAMs

In order to characterize the TAM population, we identified 30 TAM clusters by using X-shift and TSNE, taking the positive expression of CD68 and CD11b as markers (Fig. 4A). M1-like TAMs (CD163^−^) and M2-like TAMs (CD163^+^) were defined according to the expression level of CD163. The proportion of M2-like macrophages (CD163^+^CD204^+^CD206^+^) was significantly increased in tumor tissues, while the proportion of M1-like macrophages (CD163^−^CD14^−^CD206^+^) was decreased in tumor tissues (Fig. 4B-D, Supplementary Fig. 3). Among the 30 TAM clusters, only the M7 cluster was enriched in tumor tissues, which indicates that this cluster may be closely related to tumor malignancy (Fig. 4E).

In 80% of tumors, at least 10% of myeloid cells are PD-L1. However, our data showed that TAMs almost all expressed CD38 instead of PD-L1. The expression of CD38 is related to immunosuppressive macrophages in patients with renal clear cell carcinoma and MDSC-mediated T cell suppression in colorectal cancer [27]. These data indicated that in urothelial carcinoma, the immunosuppressive microenvironment regulation of TAMs did not depend on the PD-L1 pathway, and may be more mediated by the CD38 pathway.

To investigate the function of CD38^+^ TAMs, we performed GO enrichment analysis and Pathway analysis on the differentially expressed genes (DEG) of CD38^+^ TAMs and CD38^−^ TAMs in single-cell sequencing data published by *Zhaohui Chen*(v) (Fig. 4F and G, Supplementary Fig. 4) [28]. As shown in Fig. 4G, oxidative phosphorylation, respiratory electron transfer, and NADH metabolic pathways were significantly enriched in CD38^+^ TAMs, which may be related to CD38 being an NAD + -consuming enzyme.

### Correlation analysis between TAMs and T cells

**Fig. 5:**
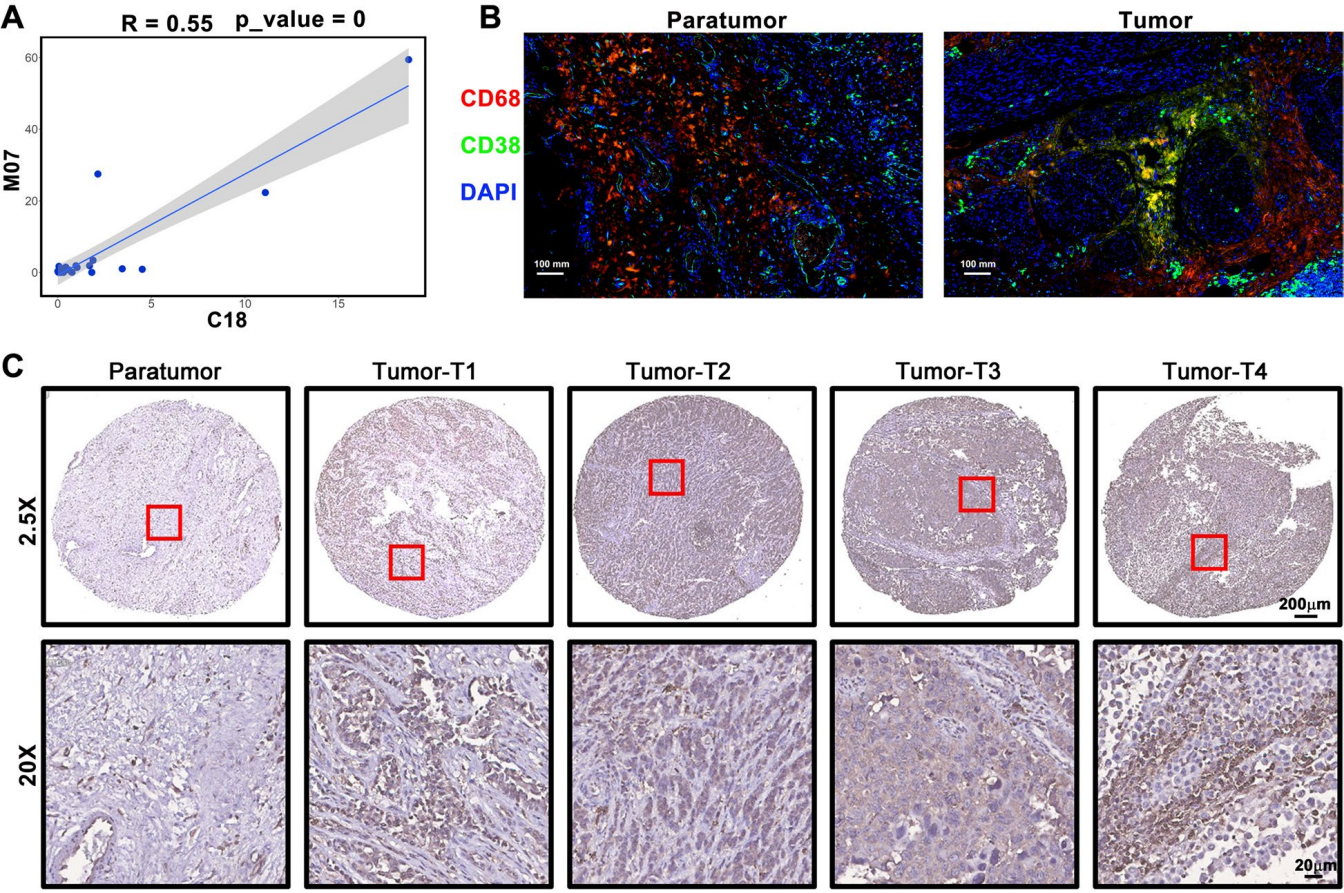
Correlation analysis between TAMs and T cells. **A** Correlation between the M7 TAM cluster and C18 T cell cluster. **B** Representative bladder cancer tissue stained for CD68 (pink), CD38 (green), DAPI (blue). Scale bar 100 µm. **C** Immunohistochemical examination of CD38 expression in paratumor tissue and different stage tumor tissue from patients with bladder cancer

For systematic quantification of the relationships between immune cell populations in TME, we calculated the percentages of each immune cell phenotype for each tumor and performed a correlation analysis based on these percentages to eliminate outlier effects (Supplementary Fig. 5). T cell clusters (C17, C18, C46, C47, C68, C70) and TAM clusters (M7) were enriched in tumor tissues. Correlation analysis showed that the M7 cluster and C18 cluster have a strong correlation (*R* = 0.55) (Fig. 5A), which indicated that the M7 cluster was involved in tumor immunosuppression regulation. The association between the two phenotypes was CD38. Immunofluorescence images confirmed that CD38 was expressed on CD68^+^ cells (Fig. 5B). We examined CD38 expression in bladder cancer tissues at various stages and the results showed that CD38 was abnormally highly expressed in tumor tissues and was closely associated with tumor progression (Fig. 5C). These data indicate that CD38 may be a potential target for the treatment of urothelial carcinoma.

### Anti*-*CD38 antibody suppresses bladder tumor growth *in vivo*

**Fig. 6:**
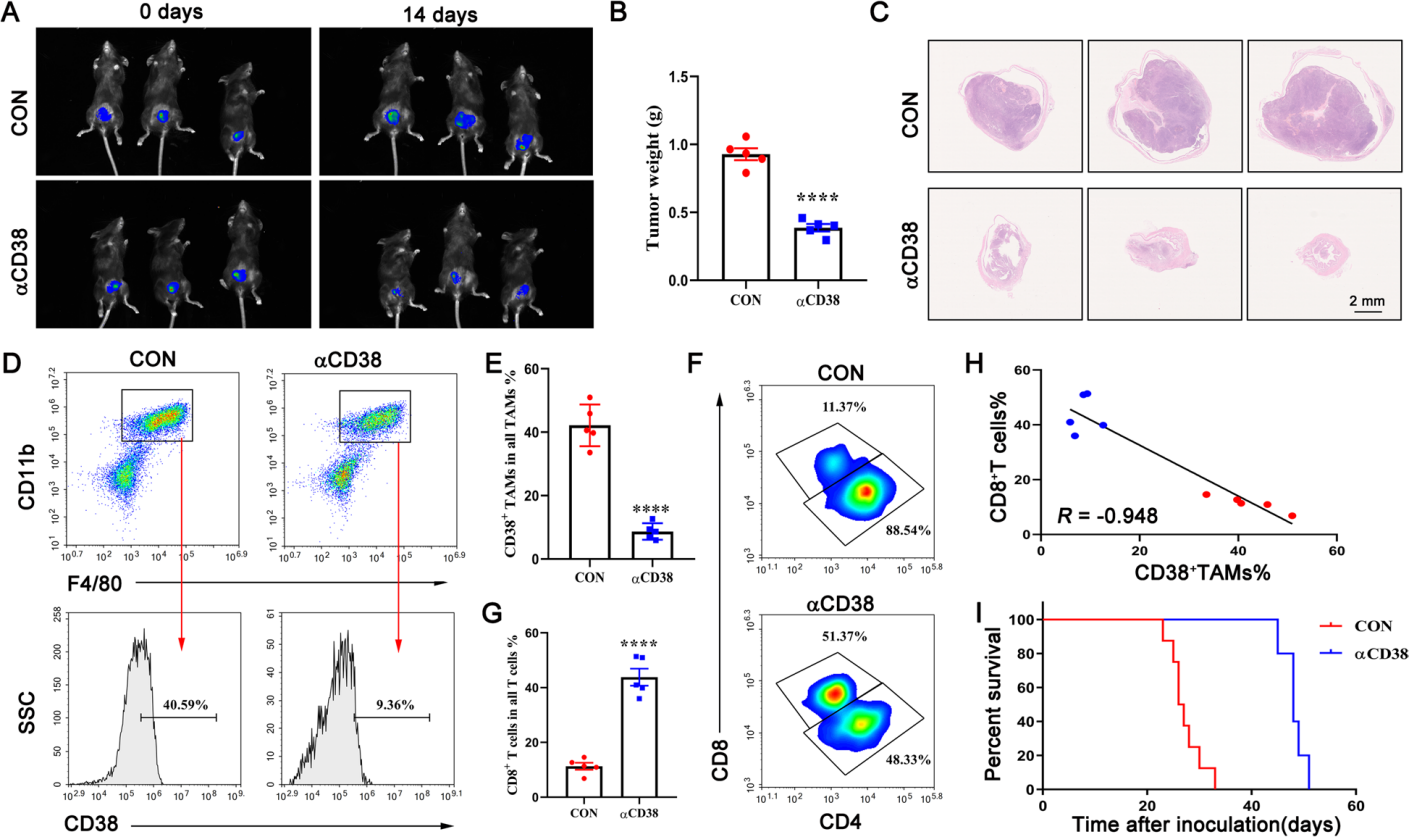
Anti-CD38 antibody suppresses bladder tumor growth *in vivo*. **A** In vivo imaging of luciferase-expressing MB49 tumor-bearing mice after intravenous injection of IgG2a isotype control antibody or anti-CD38 monoclonal antibody. **B** The weight of tumors from IgG2a isotype control antibody or anti-CD38 monoclonal antibody -treated mice was analysed. Each symbol indicates one mouse (*n* = 5). **C** The H&E images of tumors from IgG2a isotype control antibody or anti-CD38 monoclonal antibody-treated mice. **D-E** Flow cytometric analysis and quantification of tumour-infiltrating CD38^+^ TAMs (*n* = 5). **F-G** Flow cytometric analysis and quantification of CD8^+^ T cells. **H** Correlation analysis between the percentage of CD38^+^ TAMs and the percentage of CD8^+^ T cells. *R* values by linear regression. **I** Kaplan–Meier survival analysis for the overall survival of IgG2a isotype control antibody or anti-CD38 monoclonal antibody-treated mice

To test whether CD38 is the target for the treatment of urothelial carcinoma, we establish the MB49 subcutaneous xenograft model in C57BL/6 mice. Seven days after establishment of the bladder orthotopic tumor model (day 0), the bladder tumor was confirmed by the animal bioluminescence imaging instrument. Then mice were intravenously injected with IgG2a isotype control antibody or anti-CD38 monoclonal antibody. The results showed that anti-CD38 antibody treatment significantly inhibited the growth of bladder tumors compared with IgG2a isotype control antibody at day 14 (Fig. 6A, B). The antitumor effect was analyzed by histological examination of tumor tissues. H&E staining of the bladder cancer showed a decrease in tumor volume (Fig. 6C).In addition, multi-colour flow cytometric analysis confirms that treatment with anti-CD38 antibodies reduces the proportion of infiltrating CD38^+^ TAMs (Fig. 6D-E) and increased infiltration of CD8^+^ T cells in bladder cancer tissue (Fig. 6F-G). Correlation analysis revealed that the proportion of CD38^+^ TAMs was negatively correlated with the proportion of CD8^+^ T cells (Fig. 6H), which was consistent with the CyTOF data. Anti-CD38 antibody significantly prolongs survival time in tumor-bearing mice (Fig. 6I). In conclusion, these data demonstrate the importance of CD38 for tumor progression and suggest CD38 as a treatment for uroepithelial carcinoma.

## Discussion

The immune microenvironment plays a key role in tumor development, progression and prognosis. With the success of immune checkpoint blockade therapies, immune cells as a part of the complex immune microenvironment have become the focus of cancer research and drug development. Using CyTOF technology, we aimed to analyze the immune cell phenotype and improve the understanding of TME in urothelial carcinoma. We collected 12 urothelial carcinoma tissues and their corresponding paraneoplastic tissues, including 4 ureteral cancer, 3 renal pelvic cancer, and 5 bladder cancer. By using 42 markers, we present a single-cell view of the complex immune microenvironment of urothelial carcinoma, providing more insights into the immune landscape of TME.

In our study, we observed that T cell populations exhibit a complex diversity according to surface markers and that these cell populations are mainly characterized by the combinatorial expression of immunosuppressive receptors. T cells widely expressed PD-1, while LAG3, TIM3, OX40, and 4-1BB were expressed at relatively low levels, which indicates that targeting these molecules in urothelial carcinoma may be less effective than targeting PD-1. According to the expression of inhibitory receptors in patients, it is necessary to develop specific targeted therapeutic combinations to achieve precise individualized treatment and improve patient prognosis. The proportion of tissue-resident CD8^+^ T cells with high PD-1 expression in tumor tissues was significantly higher than in paratumor tissues, and this cell population also highly expressed CD38. CD38 was identified as a potential marker of T cell exhaustion by CyTOF in clear cell renal and breast cancers [17, 29]. In urothelial carcinoma, CD38 is more widely expressed than PD-1, suggesting that targeting CD38 may be more effective than targeting PD-1.

Tumor-associated macrophages are the central regulator of tumor immunity and immunotherapy [15]. Based on the antibodies we used, we identified 30 major TAM subsets with different phenotypes. CD163, CD204, and CD206 are widely used to define TAM subpopulations with pro-tumor phenotypes [16, 30, 31]. Although the expression levels of CD163, CD204, and CD206 were significantly upregulated in cancer tissues compared to paratumor tissues, CD163, CD204, and CD206 were not expressed at high levels in TAMs than other markers. More interestingly, TAMs in urothelial cancer tissues barely expressed PD-L1, which also predicts that TAMs exert immunosuppressive functions through other pathways. Different TAMs phenotypes have been shown to play different roles in tumors. Dissecting the function of TAMs subpopulations, which may be necessary to design therapeutic strategies for targeting immunosuppressive TAMs populations and to improve the efficacy of immunotherapy.

We investigated the relationship between TAMs and T cells in an attempt to provide insight into the immune function of these TAMs populations. We observed a strong correlation between the C18 T cell cluster and the M7 TAM cluster, both of which were significantly increased in tumor tissue and, more importantly, both of which were highly expressed in CD38. Studies have reported that targeting CD38 attenuates tumor progression [32]. CD38 has dual functions as an ectoenzyme and as a surface receptor and is responsible for the activation and proliferation of immune cells [33]. CD38 was found to play an important role in the immunosuppressive function of myeloid-derived suppressor cells (MDSC) in an esophageal cancer model. Although targeting CD38 antibodies has been approved for treatment of multiple myeloma [34], the role of CD38 in solid tumor, especially urothelial carcinoma, has not been investigated. It has been reported that CD38-mediated immunosuppression is a major mechanism underlying resistance to PD-1/PD-L1 blockade [26]. Deeper analysis about co-expression between CD38 and immune checkpoint molecules provides new insight to expand and improve the efficacy of immune checkpoint blockading in cancer treatment. Our results found that CD38 is more widely expressed than PD-1/PD-L1 in urothelial carcinoma, which may be one of the reasons why bladder cancer, with high mutational burden, has a low response rate to anti-PD-1 antibody therapy. We further found that CD38 expression was positively correlated with bladder cancer progression, further suggesting that CD38 may be a potential therapeutic target.

Our research also has limitations. First of all, the results of CyTOF depend on the choice of markers. The antibodies used limit the phenotypic analysis of immune cells in the tumor microenvironment. In addition, the combination of other histological techniques such as single-cell transcriptome sequencing and spatial transcriptome will enable to more adequately map the tumor immune landscape. Second, more cases are needed to establish the association between the immune microenvironment of urothelial carcinoma and patient prognosis. We acknowledge that the small number of samples used in the study, although also combined with single-cell transcriptome sequencing results from other studies, may have led to a lack of sufficient power to confirm the results we have observed at present. Third, more follow-up studies are needed to clarify the function of each immune cell subpopulation phenotype.

We performed a refined analysis of immune cells in urothelial carcinoma TME, enriching TAM and T cell phenotypic diversity and showing correlations between different cell subpopulations. Immunotherapy is an emerging therapeutic approach with great potential in recent years, especially immune checkpoint inhibitor therapy, but it is only suitable for some patients. Little is known about the mechanisms of therapeutic failure, so mapping the immune microenvironment of tumors and studying the relationships between immune cells will provide basic data to support improved immunotherapy.

## Supplementary Information

**Additional file 1:** **Supplementary Table 1.** Summary of the characteristics of 14 patients. **Supplementary Table 2.** Summary of the metal-conjugated antibodies used in the study. **Supplementary Figure S1.** Gating strategy to identify T cells and TAMs. **Supplementary Figure S2.** T cell characteristics of urothelial carcinoma, related to figure 3. **Supplementary Figure S3.** TSNE visualization showing the normalized expression of indicated markers in tumor tissues and paratumor tissues. **Supplementary Figure S4.** Pathway analysis of on the upregulated and downregulated DEGs of CD38+TAMs and CD38-TAMs. **Supplementary Figure S5.** Heatmap showing Spearman coefficients of correlation for relationships between TAMs and T cells, related to figure 5. 

## Data Availability

The datasets used and/or analysed during the current study available from the corresponding author on reasonable request.
