# Supplementary material for: Mass cytometry reveals immune atlas of urothelial carcinoma
Source: BMC Cancer. 2022 Jun 20;22:677. doi: 10.1186/s12885-022-09788-7 (PMC9210814; doi:10.1186/s12885-022-09788-7)
Supplement: Supplementary file 1 — Additional file 1: Supplementary Table 1. Summary of the characteristics of 14 patients. Supplementary Table 2. Summary of the metal-conjugated antibodies used in the study. Supplementary Figure S1. Gating strategy to identify T cells and TAMs. Supplementary Figure S2. T cell characteristics of urothelial carcinoma, related to figure 3. Supplementary Figure S3. TSNE visualization showing the normalized expression of indicated markers in tumor tissues and paratumor tissues. Supplementary Figure S4. Pathway analysis of on the upregulated and downregulated DEGs of CD38+TAMs and CD38-TAMs. Supplementary Figure S5. Heatmap showing Spearman coefficients of correlation for relationships between TAMs and T cells, related to figure 5. [file 12885_2022_9788_MOESM1_ESM.docx]

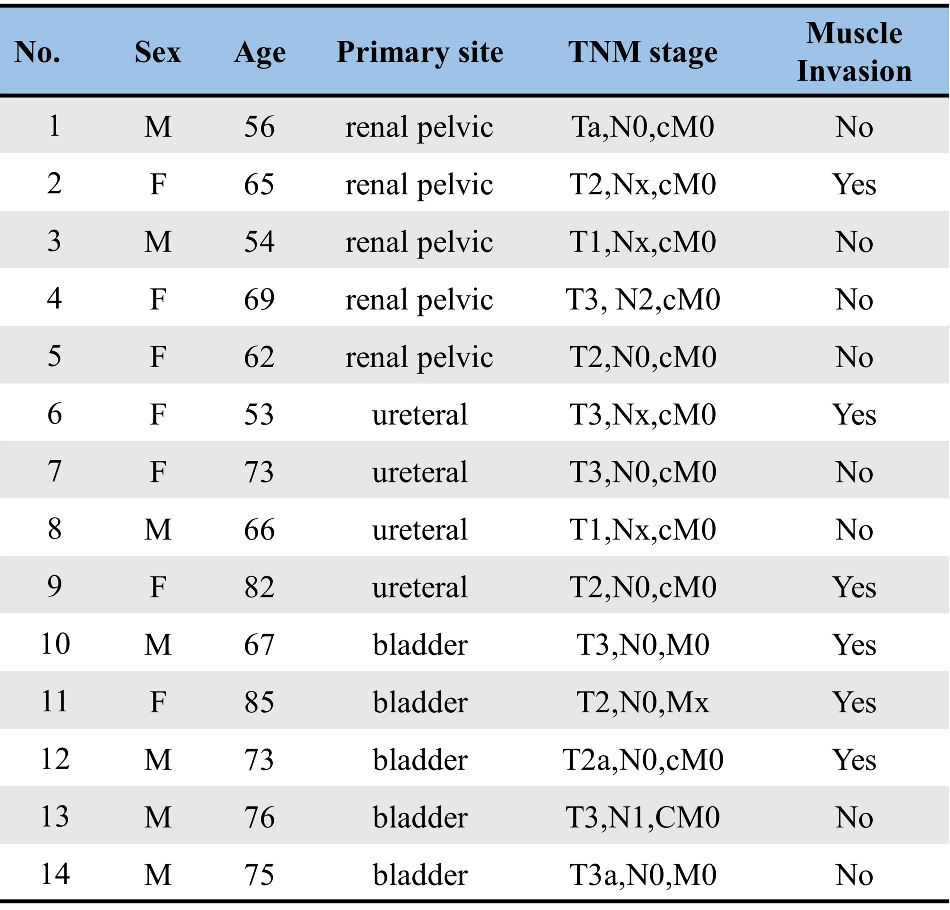


**Supplementary Table 1. Summary of the characteristics of 14 patients.**


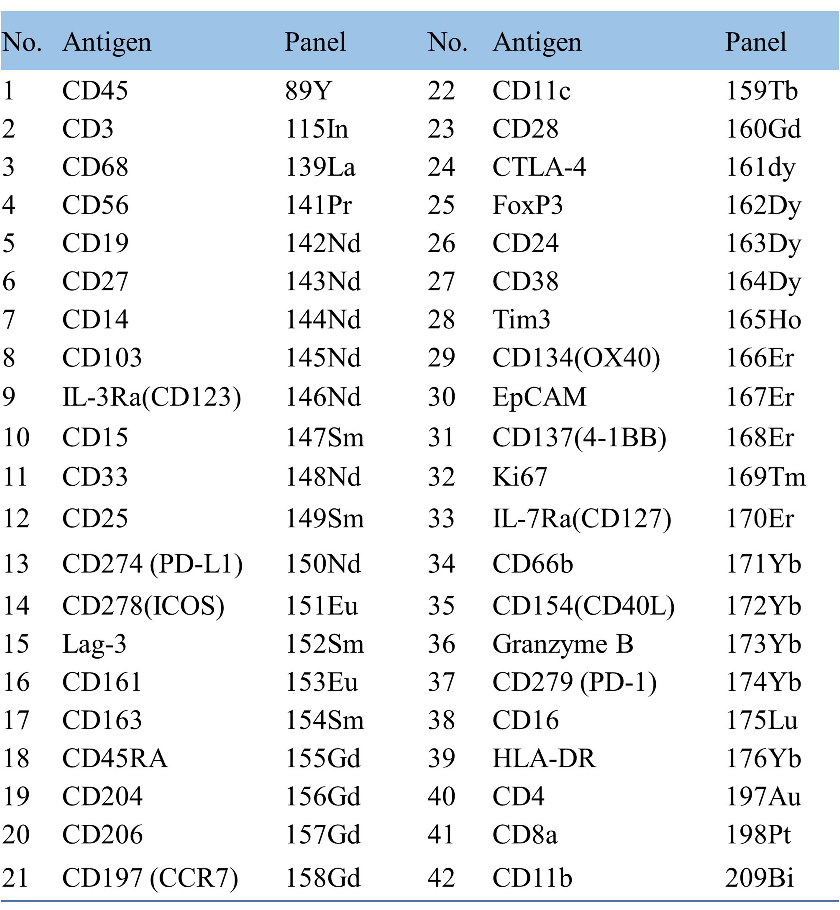


**Supplementary Table 2. Summary of the metal-conjugated antibodies used in the study.**


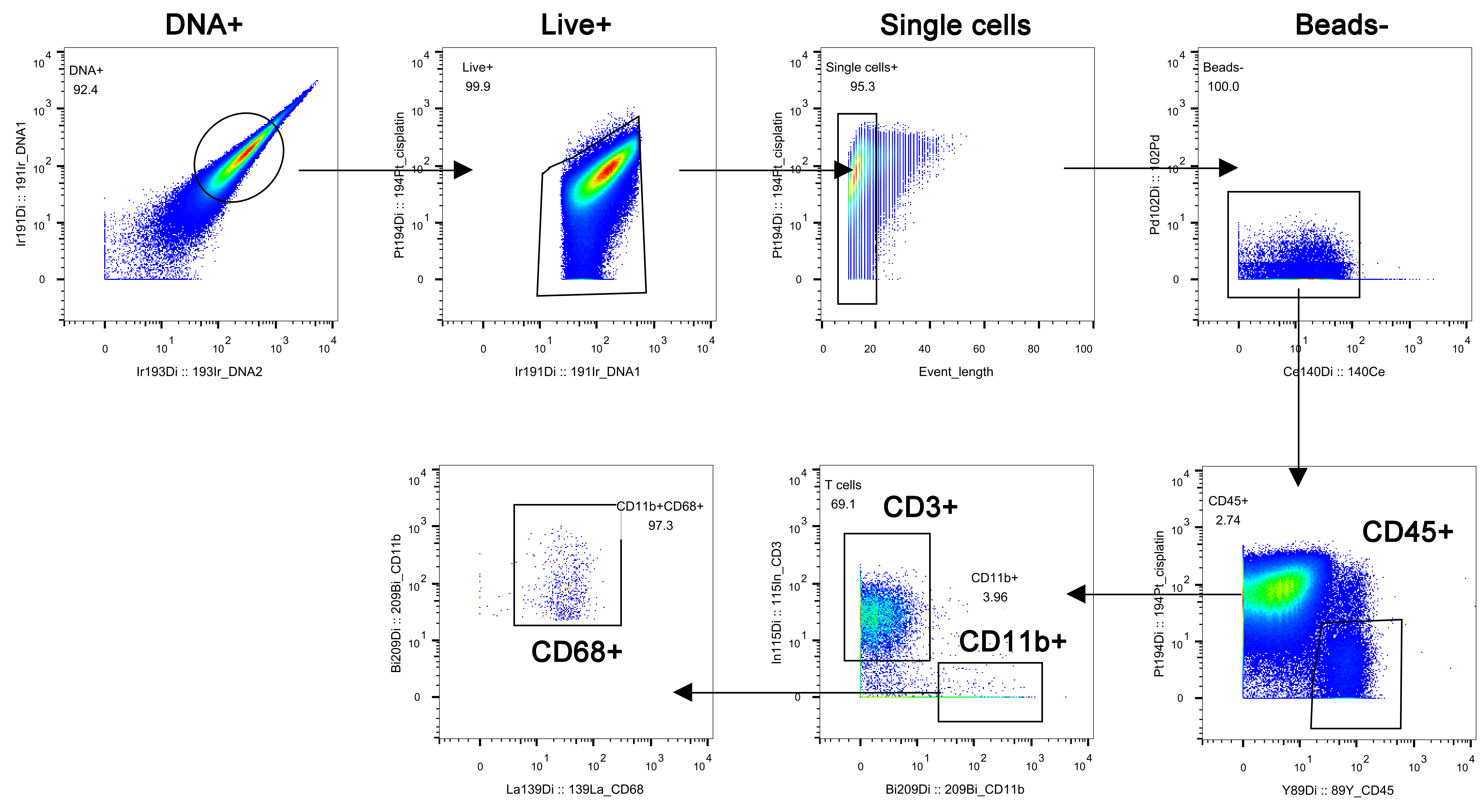


**Supplementary Figure S1.** **Gating strategy to identify T cells and TAMs.**


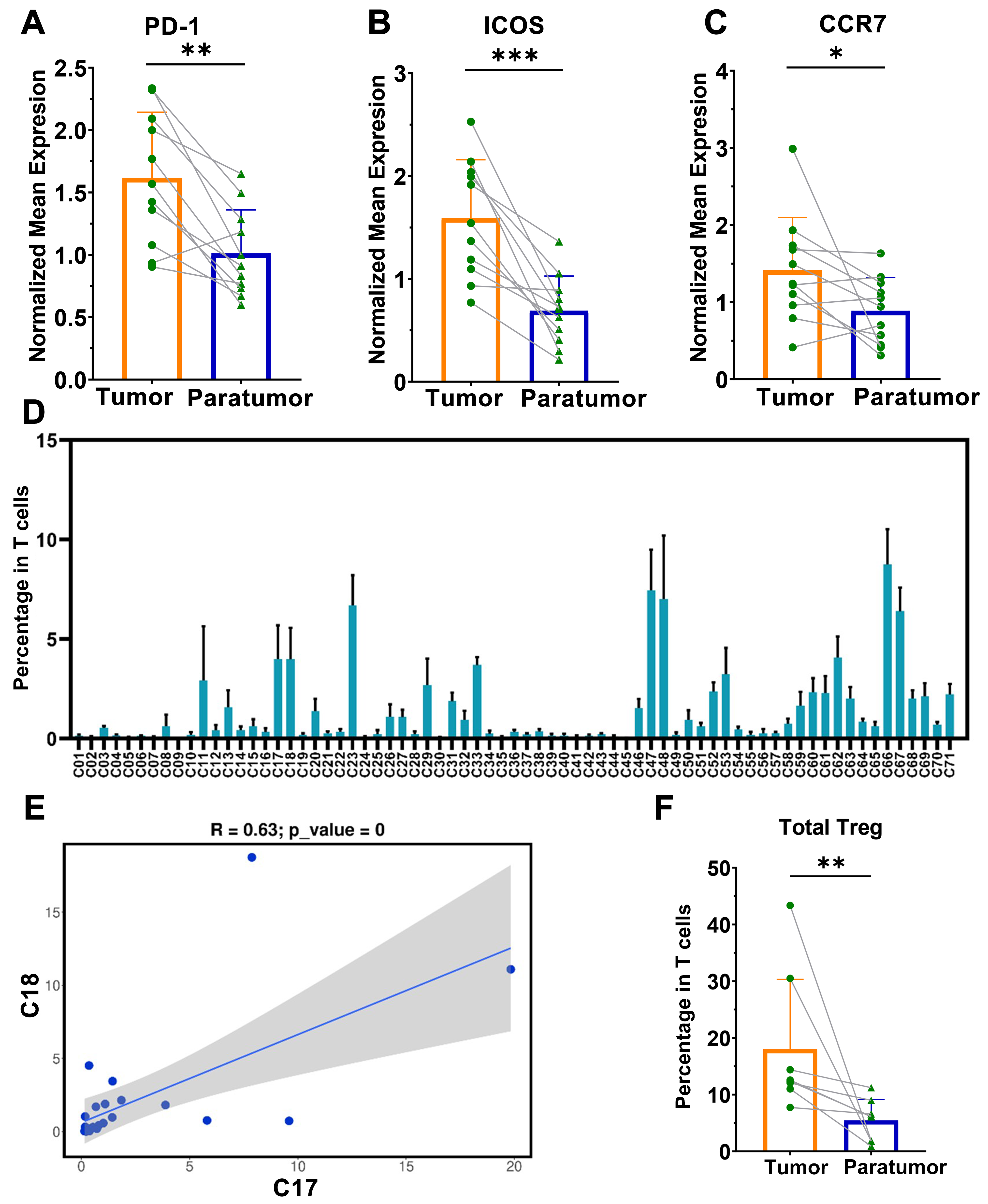


**Supplementary Figure S2. T cell characteristics of urothelial carcinoma, related to figure 3.**

**(A-C)** The normalized expression of indicated markers in tumor tissues and paratumor tissues. **(D)** Percentage of all T cell clusters in tumor tissues. **(E)** Correlation between the C17 cluster and the C18 cluster in tumor tissues. **(F)** Percentage of Treg cells in tumor tissues and paratumor tissues.


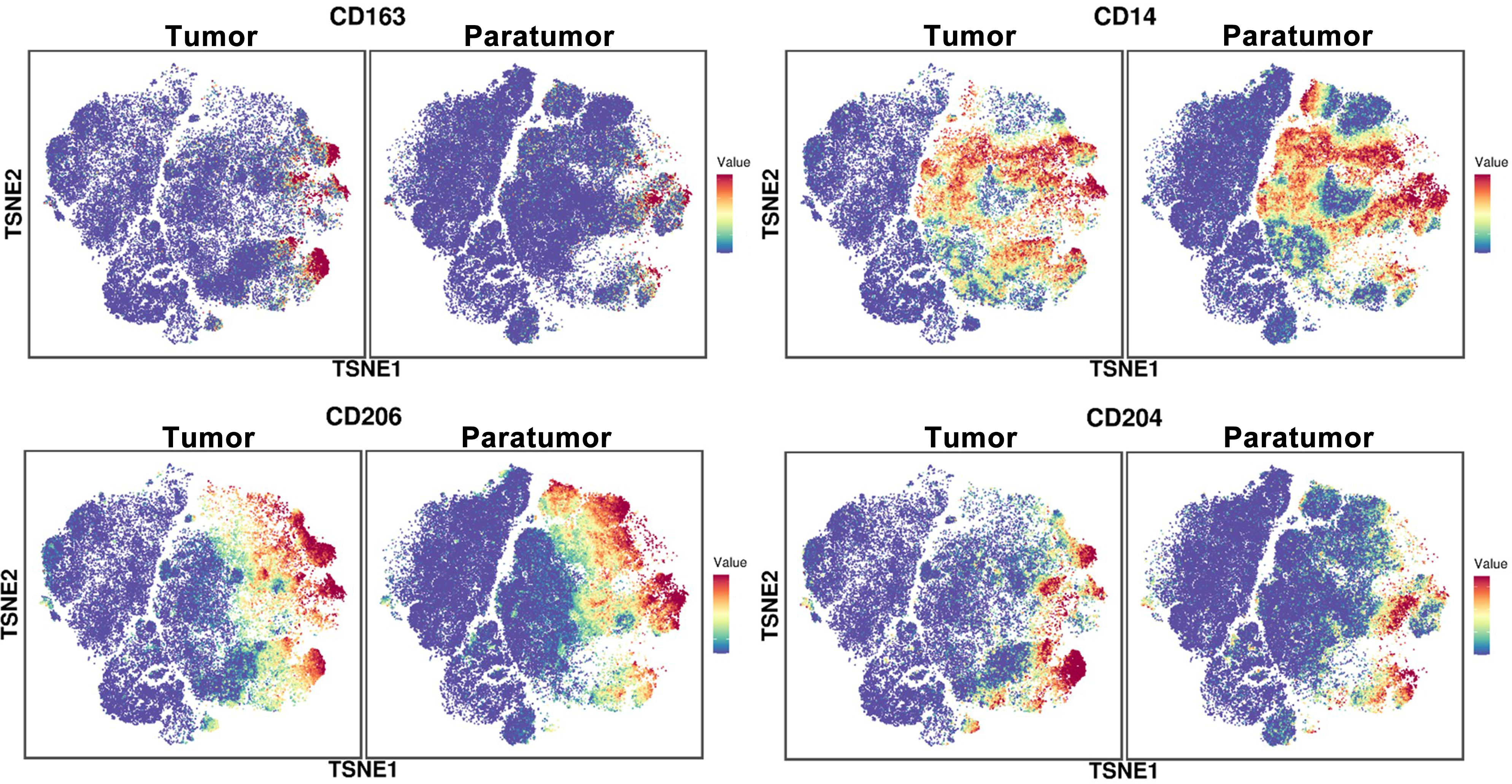


**Supplementary Figure S3. TSNE visualization showing the normalized expression of indicated markers in tumor tissues and paratumor tissues.**


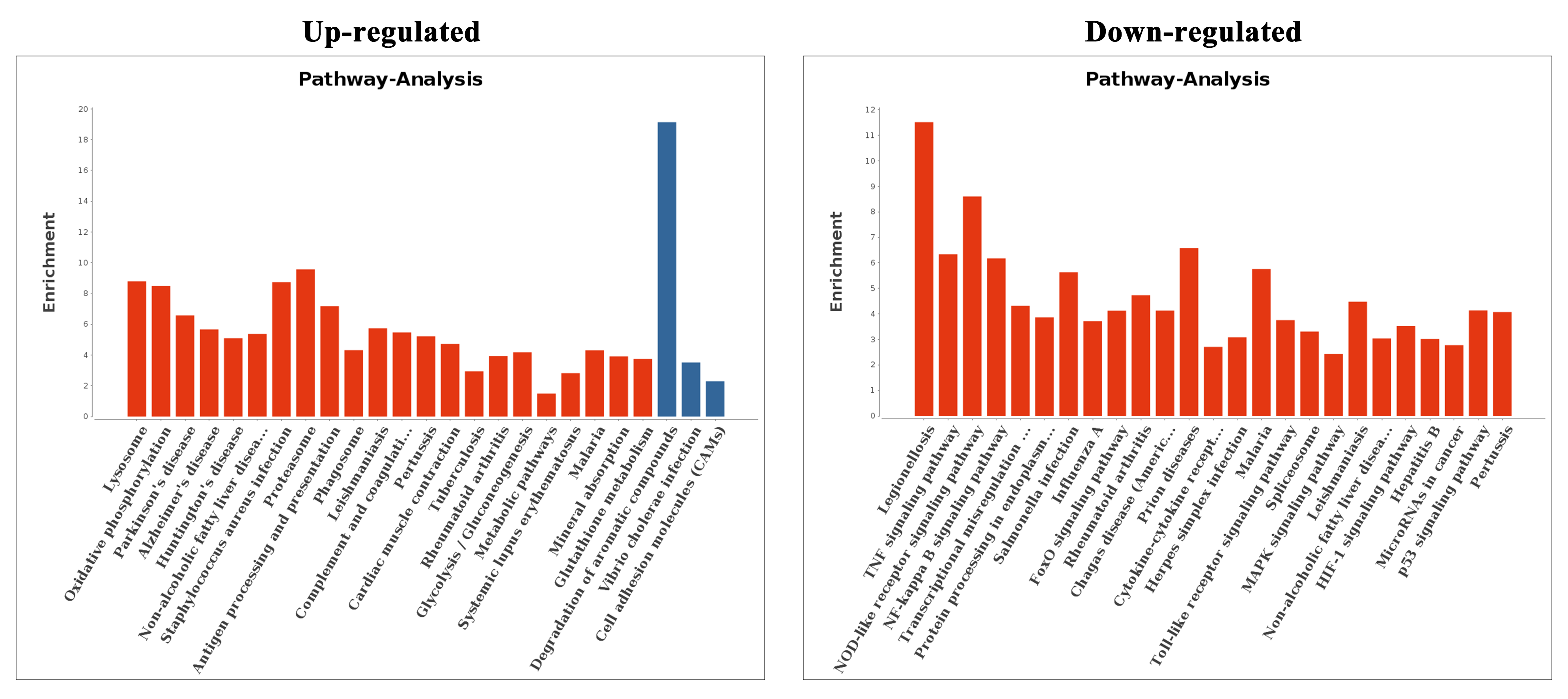


**Supplementary Figure S4. Pathway analysis of on the upregulated and downregulated DEGs of CD38+TAMs and CD38-TAMs**


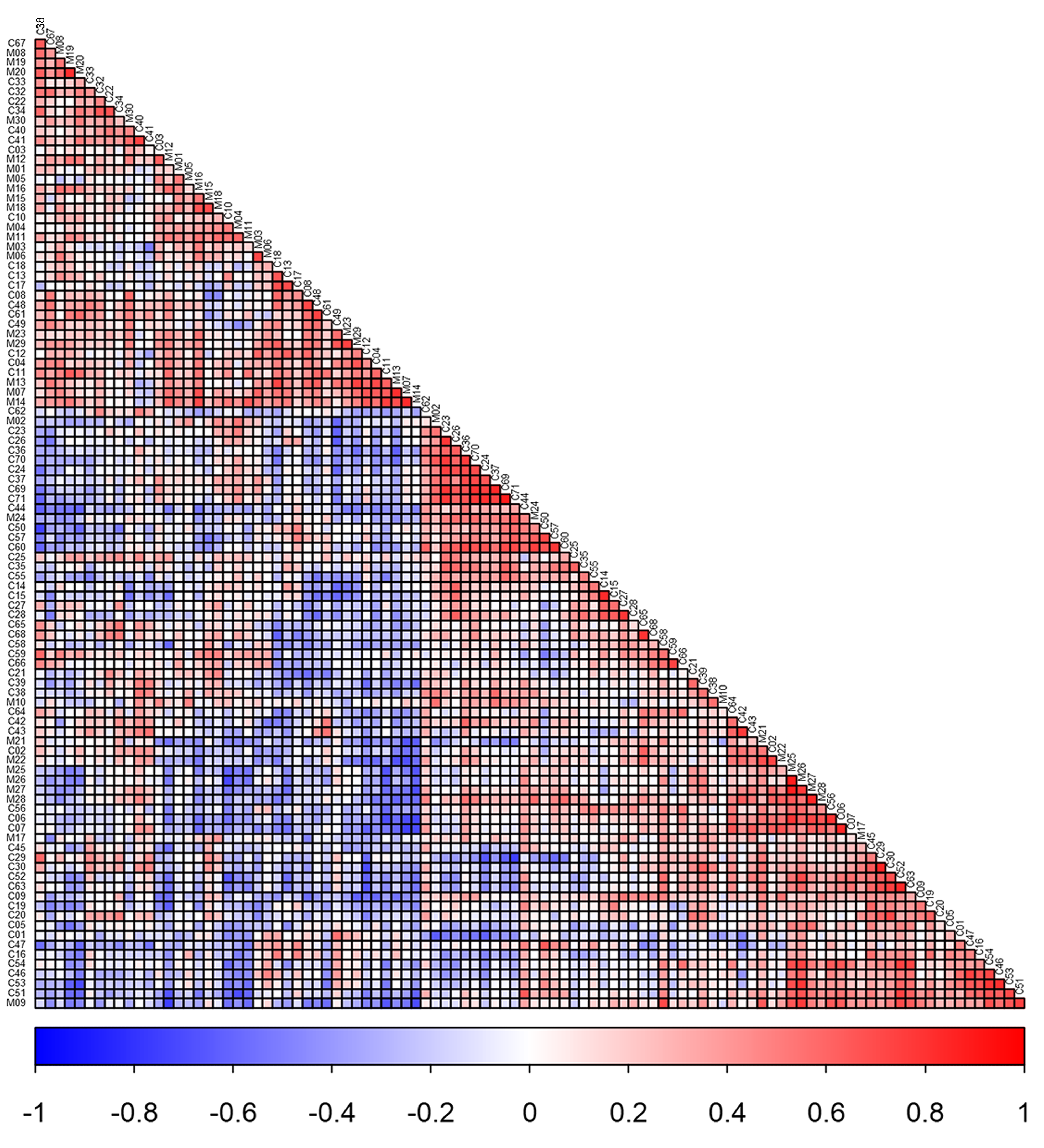


**Supplementary Figure S5. Heatmap showing Spearman coefficients of correlation for relationships between TAMs and T cells, related to figure 5**
